# High Quality Unigenes and Microsatellite Markers from Tissue Specific Transcriptome and Development of a Database in Clusterbean (*Cyamopsis tetragonoloba*, L. Taub)

**DOI:** 10.3390/genes8110313

**Published:** 2017-11-09

**Authors:** Hukam C. Rawal, Shrawan Kumar, Amitha Mithra S.V., Amolkumar U. Solanke, Deepti Nigam, Swati Saxena, Anshika Tyagi, Sureshkumar V., Neelam R. Yadav, Pritam Kalia, Narendra Pratap Singh, Nagendra Kumar Singh, Tilak Raj Sharma, Kishor Gaikwad

**Affiliations:** 1 ICAR-National Research Centre on Plant Biotechnology, New Delhi 110012, India; hukam.rawal@gmail.com (H.C.R.); kumarshrawan12@gmail.com (S.K.); amithamithra.nrcpb@gmail.com (A.M.S.V.); amolsgene@gmail.com (A.U.S.); deept_mbi@yahoo.co.in (D.N.); swatisaxena605@gmail.com (S.S.); tyagi.anshika9@gmail.com (A.T.); sureshkumarv1996@gmail.com (S.V.); nksingh@nrcpb.org (N.K.S.); trsharma1965@gmail.com (T.R.S.); 2 ICAR-Indian Agricultural Research Institute, New Delhi 110012, India; pritam.kalia@gmail.com; 3 ICAR-Indian Institute of Pulse Research, Kanpur 208204, India; npsingh.iipr@gmail.com; 4 CCS Haryana Agricultural University, Hisar 125004, India; nryadav@hau.ernet.in

**Keywords:** *Cyamopsis tetragonoloba*, clusterbean, transcriptome, RNA-Seq, tissue-specific, polymorphism, microsatellite markers, database

## Abstract

Clusterbean (*Cyamopsis tetragonoloba* L. Taub), is an important industrial, vegetable and forage crop. This crop owes its commercial importance to the presence of guar gum (galactomannans) in its endosperm which is used as a lubricant in a range of industries. Despite its relevance to agriculture and industry, genomic resources available in this crop are limited. Therefore, the present study was undertaken to generate RNA-Seq based transcriptome from leaf, shoot, and flower tissues. A total of 145 million high quality Illumina reads were assembled using Trinity into 127,706 transcripts and 48,007 non-redundant high quality (HQ) unigenes. We annotated 79% unigenes against Plant Genes from the National Center for Biotechnology Information (NCBI), Swiss-Prot, Pfam, gene ontology (GO) and KEGG databases. Among the annotated unigenes, 30,020 were assigned with 116,964 GO terms, 9984 with EC and 6111 with 137 KEGG pathways. At different fragments per kilobase of transcript per millions fragments sequenced (FPKM) levels, genes were found expressed higher in flower tissue followed by shoot and leaf. Additionally, we identified 8687 potential simple sequence repeats (SSRs) with an average frequency of one SSR per 8.75 kb. A total of 28 amplified SSRs in 21 clusterbean genotypes resulted in polymorphism in 13 markers with average polymorphic information content (PIC) of 0.21. We also constructed a database named ‘ClustergeneDB’ for easy retrieval of unigenes and the microsatellite markers. The tissue specific genes identified and the molecular marker resources developed in this study is expected to aid in genetic improvement of clusterbean for its end use.

## 1. Introduction

*Cyamopsis tetragonoloba* L. Taub (Clusterbean, 2n = 14) commonly known as guar, is a leguminous *kharif* crop grown for forage, industrial and vegetable purpose [1]. Clusterbean is a drought-tolerant legume which can survive in marginal lands including saline and low fertile soils [2,3]. In recent years, it has emerged as an industrial crop due to its gum content which serves as a raw material in a wide range of industrial applications from mining for oil and gas to food, cosmetic and textile industries [4,5,6]. Clusterbean also has medicinal value and is used in the treatment of high cholesterol and diabetics in indigenous medicine preparations [7]. The great advantage of guar gum, chemically galactomannans, compared to those of other species, such as carob (*Ceratonia siliqua* L.), is that it is extremely viscous even at low concentrations and is highly soluble in cold water [8]. Moreover, clusterbean meal, obtained as a byproduct after the extraction of gum from the endosperm is an excellent feed supplement for the livestock, broiler and fish, due to its high protein content (32–52%) [9]. India is the largest producer of clusterbean comprising of 80% of the world production [10]. India is also the major exporter of guar gum, especially to the USA, China, Germany, Russia, and Canada [9].

The major grain pulses of the sub-continent, chickpea and pigeonpea have huge genomic resources developed in the last decade [11,12,13,14]. However, clusterbean has limited genomic resources despite its huge economic importance. Due to lack of genomic resources, presently, conventional breeding is the only means of clusterbean improvement. In this regard, availability of genomic resources can serve as a good platform for clusterbean improvement. As of now, there are 78,686 sequences (62,146 unigenes, 16,476 expressed sequence tags (EST)s and 61 nucleotides sequences) available in the National Center for Biotechnology Information (NCBI) database generated from early and late developing clusterbean seeds, and leaf transcriptomes generated from two clusterbean varieties using Illumina sequencing platform [15,16]. Recently, we have published the chloroplast genome of clusterbean [17].

For precise breeding applications, availability of DNA markers is a prerequisite. Few large-scale molecular marker development efforts are reported in this crop. So far, only anonymous molecular marker systems such as inter simple sequence repeats (ISSR), random amplified polymorphic DNA (RAPD), amplified fragment length polymorphism (AFLP) and ribosomal DNA (rDNA)have been deployed in clusterbean [18,19,20,21,22]. Development of locus specific codominant DNA markers would be more appropriate for constructing high density genetic maps, marker assisted selection (MAS), evolutionary and population genetics studies of this species. Thirty-nine polymorphic simple sequence repeats (SSRs) developed from 16,476 EST sequences are the only locus specific markers available in this legume crop [23]. 

For large-scale discovery and characterization of functional genes and genome assembly, global exploration of the transcriptome is a useful strategy. Sequencing of RNA remains the gold standard for annotation of both coding and non-coding genes [24,25]. RNA-Seq method offers a holistic view of the transcriptome, revealing many novel transcribed regions, splice isoforms, genic microsatellites and the precise location of transcription boundaries [26,27,28,29]. In the present study, transcriptome analysis of three different tissues namely, leaf, shoot, and flower from clusterbean are reported which will serve as a valuable resource for whole genome assembly of clusterbean, identification of tissue specific genes and promoters, and development of molecular markers.

## 2. Materials and Methods

### 2.1. Plant Material and RNA Isolation

RGC 936, a popular, short-duration, early maturing clusterbean variety was used for transcriptome analysis. It is a branched variety bearing white flowers and round and pink colored seeds containing 33% gum. Plants were grown in 10′′ pots at the Net house of ICAR-NRCPB (Indian council of Agricultural Research-National Research Centre on Plant Biotechnology), New Delhi under natural conditions in the crop season. The plants were irrigated every alternate day with normal tap water. Leaf, shoot and flower tissues were harvested at the first flowering stage, i.e., 42 days after sowing, frozen in liquid nitrogen and kept at −80 °C till further use. Total RNA from three biological replicates for each tissue was isolated from the harvested samples using Spectrum Plant Total RNA Kit (Sigma, St. Louis, MO, USA) following the manufacturer’s protocol.

### 2.2. Library Preparation and RNA Sequencing

Total RNA was quantified using Nanodrop spectrophotometer (Thermo Scientific, Waltham, MA, USA) and the quality assessment was performed with RNA 6000 Nano assay kit using Bioanalyser 2100 (Agilent, Santa Clara, CA, USA). RNA from a single tissue from all the three biological replicates type was pooled in equi-molar concentrations. The RNA sequencing library of leaf, shoot and flower tissues were separately constructed using Truseq RNA Sample prep kit (Illumina, Singapore) following the manufacturer's protocol. The average size of library was 260 bp. The libraries were then sequenced by Illumina paired end sequencing technology. The raw sequence data for three tissues has been deposited at NCBI Short Read Archive (SRA) with accession number SRR5428802, SRR5428803 and SRR5428804.

### 2.3. RNA-Seq Data Processing and De Novo Assembly

RNA-Seq raw reads were first processed for trimming (with Phred Score 33) using Trimmomatic-0.36 to remove low quality sequences and reads shorter than 36 bp [30]. The resultant high quality trimmed reads were de novo assembled with three different assemblers including Trinity2.2.0 [31], CLC Genomics workbench 7.0 (CLC Bio, Aarhus, Denmark) and SPAdes 3.9.0 [32] at different *k*-mers. The different assemblies, so obtained, were compared for different aspects including assembled transcriptome size, transcripts number, average length and N50 length of assembled transcripts. The assembly with Trinity2.2.0 at *k*-mer 25 with normalization 30 and the minimum *k*-mer coverage of 2 was found to be the best and used in downstream analysis (Supplementary Table S1).

Further, transcripts were cleaned using perl script SeqClean (https://sourceforge.net/projects/seqclean/) to remove contaminations like rRNA, low-complexity RNA, and polyA stretches. Cleaned transcripts were clustered by CD-HIT V4.6 to remove redundancies and unigenes were obtained with sequence identity and similarity cut-off set at 97% and 95%, respectively [33]. A commonly accepted estimate for the expression level of unigenes, FPKM (Fragments per kilobase of transcript per millions fragments sequenced) was calculated by running RSEM (RNA-Seq by Expectation Maximization) module from Trinity package [34]. Unigenes with FPKM < 1 and length ≤ 200 bp were removed to avoid any potential assembly errors and to ensure the quality of the resulted assembly. The high quality (HQ) unigenes (with FPKM ≥ 1 and length > 200 bp) were used in the downstream analysis.

### 2.4. Functional Annotation of High Quality Unigenes

HQ unigenes were BLAST-searched against different databases including Annotated Plant Genes (APG) database from NCBI (22,77,559 genes), Swiss-Prot (4,64,207 genes), Pfam, gene ontology (GO), KEGG and Enzyme Commission (EC) numbers using BLASTx program with a cut-off *E*-value of 1 × 10^−10^ [35]. Functional descriptions were assigned to HQ unigenes with BLAST results against APG database using Blast2GO 4.0.2 [36]. Blast2GO was also used to perform InterproScan (IPS) Search, assign Gene ontology terms (GO) and carry out pathway analyses (using KEGG).

### 2.5. Differential Gene Expression Analysis

To perform the tissue-specific transcriptome analysis, high-quality trimmed reads from each of the three samples were mapped against the assembled transcripts using RSEM package version 1.2.31 and the abundance estimates were obtained [34]. Transcripts with FPKM value ≥ 1 in all 3 tissue samples were considered as housekeeping genes [37]. In order to identify tissue-specific genes, the expression results were further parsed based on FPKM values with X-fold higher for one tissue as compared to the FPKM values for the remaining two tissue samples for a specific gene, where X = 5, 8, 10 and 50.

To estimate the gene expression level, we categorized all assembled genes into seven different categories, at a threshold of 5-fold FPKM. “Un-expressed” are those with <1 FPKM in all 3 tissues, while “tissue specific genes” are those with at least 5-fold higher FPKM in one tissue as compared to the other two tissues. The contigs with >5 FPKM value in all the three tissues were categorized as “Expressed in all” while those in only two tissues were grouped as “Mixed expressed”. Among the contigs which had <5 FPKM, those detected in only one tissue were “Low expressed but tissue specific” while the ones detected in two tissues were, “Mixed but low expressed” and those detected in all 3 tissues but at least one tissue <5 FPKM value were “Expressed in all low”.

Using the Bioconductor package edgeR (Extraction of Differential Gene Expression R Package 3.3.1) with dispersion value as 0.1 and other parameters using default settings, differentially expressed genes (DEGs) were extracted [38]. We used a *p*-value cut-off of ≤0.05 with at least two-fold change (*C* value ≥ 1) to identify significant DEGs. For graphical illustration of the expression profiles of the identified DEGs, heatmaps and plots were generated. A clustered heatmap was generated for pairwise comparison between three tissue samples representing the Pearson correlation matrix.

### 2.6. Simple Sequence Repeat Mining

The Perl script MISA [39] (http://pgrc.ipkgatersleben.de/misa/) was used to identify simple repeat sequences (SSRs) in the assembled transcripts as well as in the HQ unigenes. The minimum number of nucleotide repeats searched during the SSR analysis was set as six for di-nucleotide repeats and five for tri-, tetra-, penta- and hexa-nucleotide repeats with maximal number of bases interrupting 2 SSRs in a compound microsatellite as 100.

### 2.7. Marker Validation and Diversity Analysis

Validation of the SSR markers was carried out using 21 clusterbean accessions, obtained from Chaudhary Charan Singh Haryana Agricultural University, Hisar, India. Genomic DNA was extracted from young leaves of these accessions using cetyltrimethylammonium bromide (CTAB) extraction method [40]. DNA quality was evaluated by 0.8% agarose gel electrophoresis. The working concentration of DNA was adjusted to 50 ng/µL, for use in marker genotyping. Amplification was performed in 20 μL volume reactions containing, PCR buffer (10 mM Tris pH 9.0, 50 mM KCl), 1.5 mM MgCl_2_, 0.6 U Taq DNA polymerase (Bangalore Genei, Bangalore, India), 2 μM of dNTP, 10 pM of primer, and 50 ng of genomic DNA. Microsatellite loci were amplified on a Thermal Cycler (Applied Biosystems Veriti, Foster City, CA, USA). PCR amplification was carried out with the following cycling conditions: one cycle of 4 min at 94 °C followed by 35 cycles at 94 °C for 30 s, 55–60 °C for 30 s and 72 °C for 30 s. The final extension was performed at 72 °C for 10 min. After completion of the amplification, 2.5 mL 6× blue dye was added to the samples, and the amplified DNA was analyzed on 3.5% MetaPhore^TM^ agarose (Lonza Rockland Inc., Rockland, ME, USA) gels in 1× TBE buffer at 120 V for 4–5 h. A 50 bp DNA ladder was also resolved to determine the approximate size of the fragments. The gel was documented in the gel documentation unit (Syngene, Cambridge, UK). Since we identified only a maximum of two alleles in polymorphic markers, Numerical Taxonomy System (NTSYS-pc) ver. 2.1 (Exeter Software, Setauket, NY, USA) was used for construction of similarity matrix using the SSR genotyping data scored as presence and absence. From the binary data matrix, Jaccard’s similarity coefficient between pairs of accessions was calculated in the SIMQUAL module. Further, using the un-weighted pair grouped method arithmetic average (UPGMA), a dendrogram depicting diversity and genetic relationship among the accessions was constructed.

### 2.8. Database Design

ClustergeneDB, a database for retrieving information on the unigenes of clusterbean was constructed by using XAMPP (Apache, MariaDB, PHP and Perl) server. Backend of the database was designed using MySQL while the front-end was designed using HTML5 and CSS3. Jquery and Javascript were used to create the user framework. PHP5 was used to connect users and server to access queries. The database is hosted in the server environment, FUJITSU PrimeRGY-Rx600S6 and Windows operating system. Microsatellites present in the unigenes and their expression profiles were also added in the relational database. The database can be accessed at http://14.139.229.201/clustergenedb.

## 3. Results

### 3.1. Transcriptome Sequencing and De Novo Assembly

To comprehensively construct the complete transcriptome of *C. tetragonoloba*, RNA from three tissues representing different development stages, including leaves, shoots and flowers were sequenced. In total, 149 million paired-end raw reads with an average read length of 100 bp were generated from three tissue samples (Table 1). After quality check, trimming of adapter sequences and size selection, 145 million HQ reads (97.3%) remained for assembly. From 22.16 Gb of trimmed reads (109 million paired reads and 36 million un-paired reads), a total of 127,706 transcripts were reconstructed into 179 Mbp with N50 of 2263 bp and the largest transcript length of 16.94 kb (Table 1 and Table 2). To reduce redundancy and potential assembly errors, we clustered assembled transcripts into 110,485 unigenes and removed transcripts with FPKM values <1 and sequence length < 200 bp, since these are more likely to be error prone. As a result, a final dataset of 48,007 HQ unigenes with an average length of 1583.43 bp and an N50 of 2179 bp was obtained (Table 2). The assembled size of these HQ unigenes accounted for 76.01 Mb. The average GC content was found to be less than 40%. The size and GC% distribution for these HQ unigenes is shown in Supplementary Figures S1 and S2, respectively. Their size distribution depicted that 63.07% of these unigenes were >1 kb long while 19.26% were 500–1000 bp long. Only 17.63% of total HQ unigenes were shorter than 500 bp. A high proportion of these unigenes (84.77%) had GC content in the range of 35 to 45%.

### 3.2. Functional Annotation of HQ Unigenes

**Figure 1:**
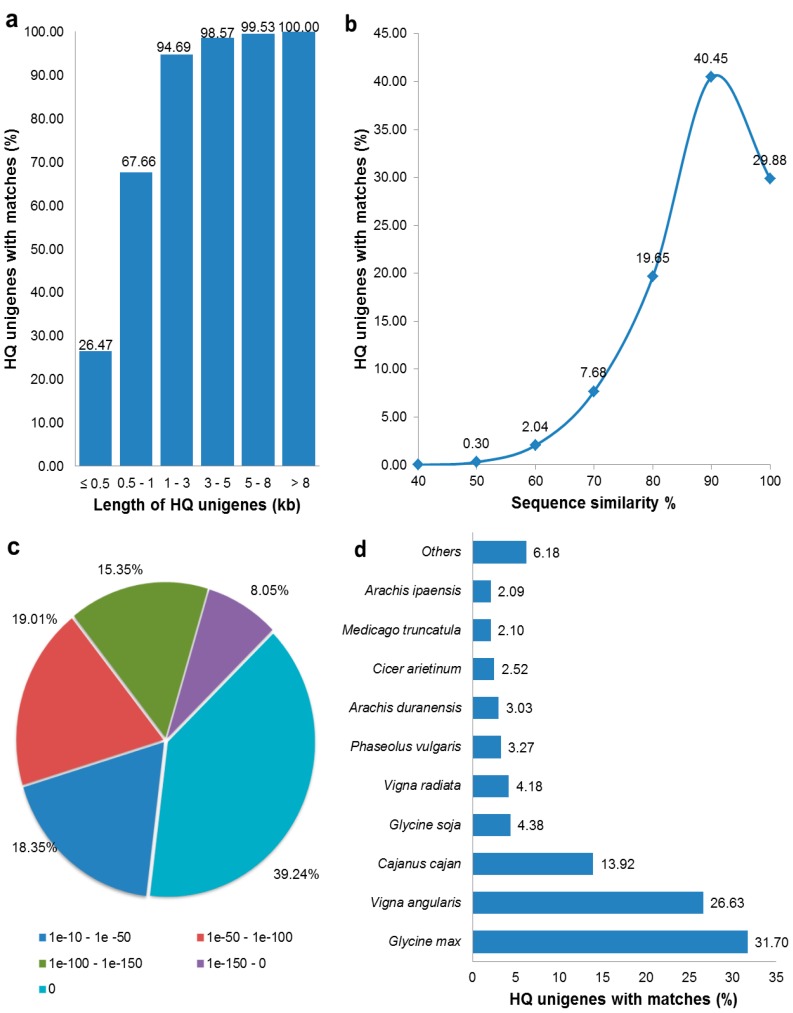
Statistics of BLAST search results of HQ Unigenes against Plant-genes database. (**a**) Length-wise distribution of HQ unigenes (query) sequence with significant matches (*E*-value ≤ 1 × 10^−10^). A very high proportion (>98%) of large unigenes (>3 kb) showing significant matches; (**b**) similarity distribution of the best BLAST hits for each of the unigene with significant matches showing that 70.33% these having sequence similarity from 80 to 100%; (**c**) percent distribution of HQ unigenes on the basis of their *E*-values; (**d**) species distribution showing percentage of the HQ unigenes (query) sequence with significant matches against different species with maximum (31.70%) of these were having top BLAST hits against *Glycine max.*

A total of 37,382 unigenes (77.87%) were found to show significant matches against downloaded plant proteins with known functions with top BLAST hits having *E*-value cut-off ≤ 1 × 10^−10^ (Table 2). As the efficiency of BLAST hits can be judged best with the length of query transcripts sequences [41], we plotted length of unigenes against the percentage of unigenes with significant matches. We found a very high proportion of matching efficiency for longer assembled unigenes (98% for >3 kb long transcripts; and 94.69% for transcripts between 1 kb and 3 kb long), however only 26.47% of shorter transcripts (≤500 bp) could find a match (Figure 1a). Similarity distribution of the best BLAST hits revealed that 70.33% the unigenes with significant matches had sequence similarity in the range of 80% to 100% while 29.37% unigenes had 50% to 80% similarity (Figure 1b). *E*-value distribution showed that 62.64% of the HQ unigenes had higher homology with top BLAST hits with small *E*-values (≤1 × 10^−100^) including 39.24% of unigenes which had perfect *E*-value of 0 (Figure 1c). Species distribution of BLAST results showed that 31.70% of unigenes with significant matches had top BLAST hits against *Glycine max,* followed by *Vigna angularis* (24.45%), *Cajanus cajan* (13.92%), and other legumes with less than 5% matches (Figure 1d). Further, 28,905 unigenes (60.21%) showed significant (*E*-value ≤ 1 × 10^−10^) BLAST matches with Swiss-prot compared to that of 77.87% with APG database (Table 2). On comparison, we found that there were 28,845 unigenes (60.08%) with hits in both of these two databases. Finally, we obtained 37,442 unigenes (77.99%) with significant hits against any of these databases, which were considered as the annotated genes of *C. tetragonoloba* transcriptome (Table 2).

### 3.3. Functional Classification with Gene Ontology Terms, Enzyme Commission Numbers and InterproScan Search

**Figure 2:**
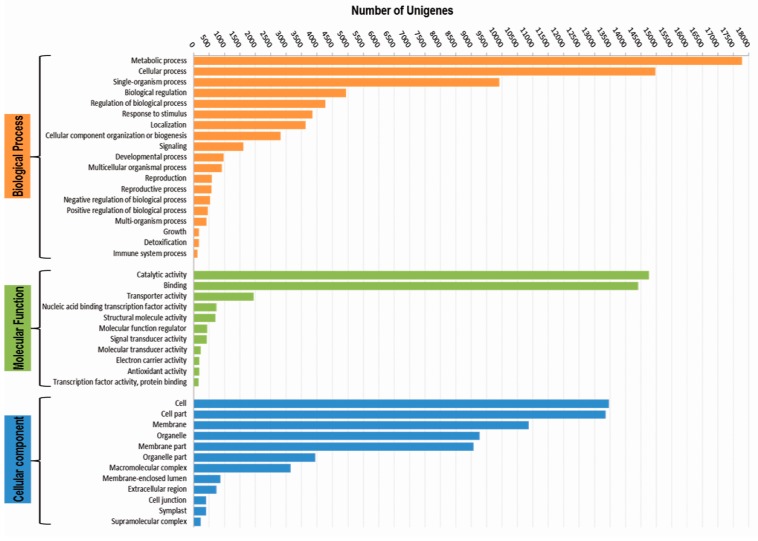
Functional classification of unigenes based on GO terms, showing GO category distribution of unigenes at GO level 2 into 3 categories: Biological Process, Molecular Function and Cellular Component.

Out of the 37,382 unigenes, 30,020 unigenes were assigned GO terms while the remaining 9984 were assigned EC numbers (Table 2). A total of 116,964 GO terms were assigned to 30,020 unigenes and their distribution at GO level 2 is shown for molecular functions, biological processes and cellular components in Figure 2. Based on assigned GO terms, unigenes were found to be highly enriched in “cellular process” and “metabolic process” under biological process classification, “catalytic activity” and “binding” under the molecular function classification, and “cell” and “cell part” under the cellular component classification (Figure 2). Out of 9984 unigenes with EC numbers, maximum number of unigenes encoded hydrolases followed by transferases and oxidoreductases (Supplementary Figure S3). Investigation of the biological pathways identified a total of 6111 unigenes sequences mapped to 137 KEGG pathways.

**Figure 3:**
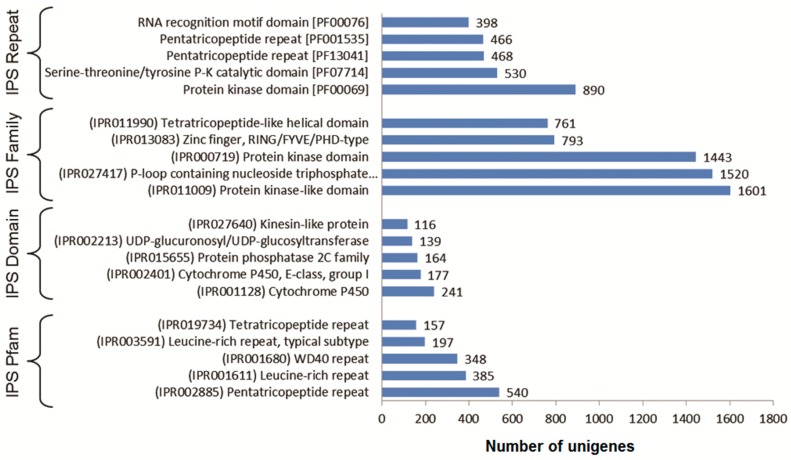
Result showing top 5 hits against interproscan repeat, family, domain and Pfam database.

InterproScan search revealed that maximum numbers of hits were found for Pentatricopeptide repeat (540 seq), Cytochrome P450 (241 seq) and Protein kinase-like domain (1601 seq) under the IPS repeat, family and domain category, respectively. Protein kinase domain [PF00069] showed maximum hits for IPS search against Pfam Database with 890 seq, followed by Serine-threonine/tyrosine protein kinase catalytic domain [PF07714], Pentatricopeptide repeat [PF13041], Pentatricopeptide repeat [PF001535] and RNA recognition motif domain [PF00076] (Figure 3).

### 3.4. Tissue-Specific Transcriptome Analysis

**Figure 4:**
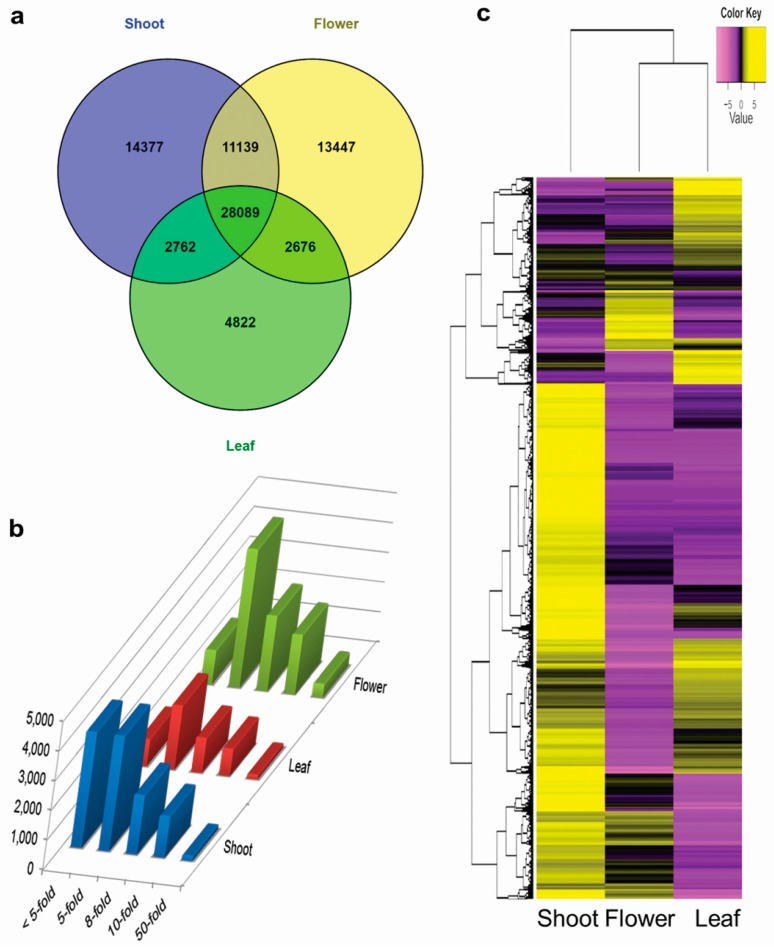
Tissue specific expression of *C. tetragonoloba*. (**a**) Expressed genes (FPKM ≥ 1) in 3 sample tissues; (**b**) expression of genes at different folds, with at each fold level of 5 or higher FPKM value, genes were found expressed higher in flower tissue sample (reproductive stage) as compared to tissue of vegetative stage (shoot and leaf); (**c**) differentially expressed genes (DEGs) vs. samples Heatmap showing cluster analysis of 38,423 differentially expressed genes for tissue-specific expression in all the 3 tissues. DEGs partitioned into 10 gene clusters with similar expression patterns with genes in each cluster ranging from 3755 to 3940. Color scale representing normalized expression values (left-top).

A total of 28,089 genes were identified as housekeeping genes with FPKM value ≥1 in each of the three tissue samples which included 12,754 (45.40%) genes with the highest FPKM value in shoot tissues (Figure 4a). At all fold level of comparisons namely 5, 8, 10, and 50, genes were found to be expressed higher in flower tissue sample (reproductive stage) followed by shoot and leaf (vegetative stage) (Figure 4b, Supplementary Table S2). A total of 790 genes were found to be highly tissue enriched with 50-fold higher FPKM value in one tissue compared to other tissues. Among these 790 highly tissue enriched genes, 58.48% were floral tissue specific followed by 22.15% and 19.37% in shoot and leaf respectively. Of the total 127,706 transcripts, a total of 50,394 were found “un-expressed” with <1 FPKM in all 3 tissues. Among the expressed genes (at a threshold of 5-fold FPKM), 16,650 were found to be tissue specific genes while 10,678 were “genes with mixed-expression” (expressed in two tissues, whether low or high) and 49,984 as “Expressed in all” (expressed in all 3 tissues, whether low or high) which accounts for 21.54%, 13.81% and 64.65% of total expressed genes, respectively (Supplementary Table S3). Out of these, a small set of randomly selected genes from “Expressed in all” tissue-group was validated with quantitative real time PCR (qRT-PCR) and observed similar expression pattern (Supplementary Figure S4).

### 3.5. Differential Expression of Genes

A total of 38,423 Differentially Expressed Genes (DEGs) were identified at a *p*-value cut-off of ≤0.05 with at least two-fold change (*C* value ≥ 1). MA and Volcano plots of DEGs for Flower vs. Leaf, Flower vs. Shoot and Leaf vs. Shoot comparison are given in Supplementary Figure S5 wherein significant differential expression is represented as red dots while genes with ”no significant expression” is represented by black ones. A significant global and relative relation between the floral and vegetative tissue as well as between the two vegetative tissues was found with positive correlation coefficient of 0.446, 0.435, and 0.401 (Supplementary Figure S6). Cluster analysis partitioned the DEGs into 10 gene clusters, each cluster having similar expression and comprising of 3755 to 3940 genes. The clustered heatmap suggested that flowers followed a contrasting transcriptomic profile to leaves, while shoots showed totally different profile from these two tissues (Figure 4c).

### 3.6. Simple Sequence Repeat Prediction 

From the assembled transcripts and HQ unigenes, we obtained a total of 17,593 and 8687 SSRs with an average frequency of one SSR per 10.20 and 8.75 kb in assembled transcripts and HQ unigenes, respectively (Table 3). Out of total 48,007 HQ unigenes, 7047 (14.68%) were found to contain SSR and 1297 of these unigenes had more than one SSR with 590 of these present in compound formation. The most abundant class of repeat motifs was found to be of those trinucleotide (51.11%) followed by dinucleotides (43.10%) SSRs. Other repeat motifs were just a fraction of these amounting to 4.43%, 0.71% and 0.64%, of tetra, penta and hexanucleotide repeats respectively (Supplementary Tables S4 and S5).

### 3.7. Marker Validation

**Figure 5:**
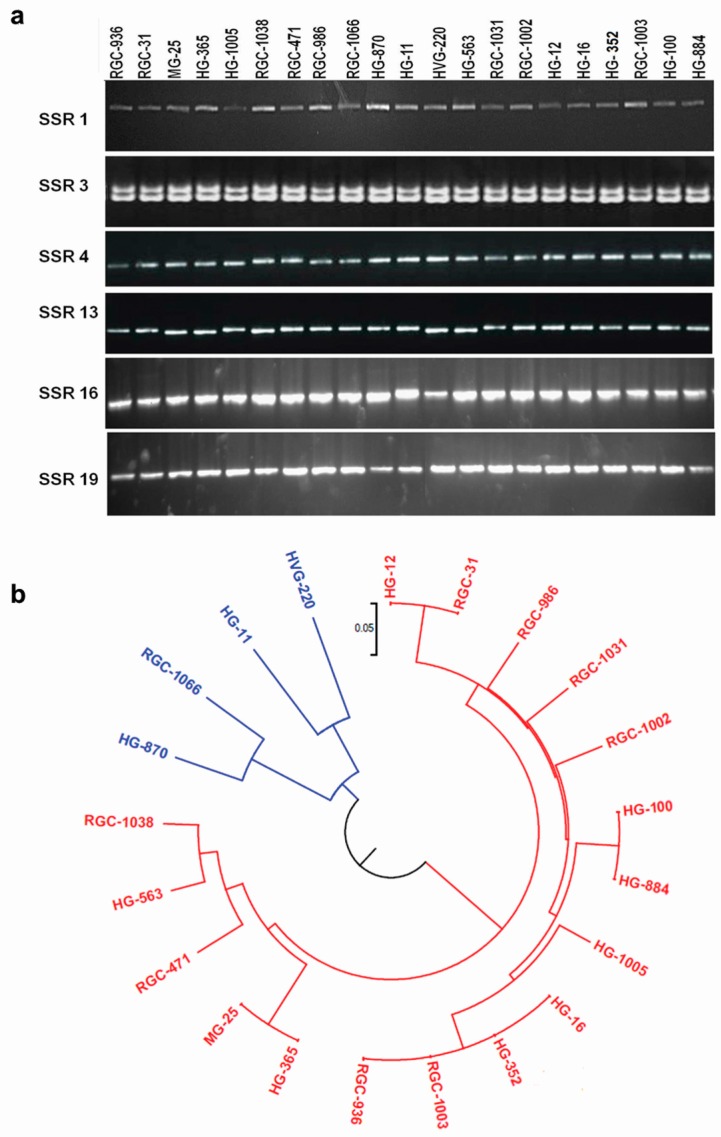
Validation of randomly selected simple sequence repeats from HQ unigenes. (**a**) Banding pattern of SSR primers’ amplification on genomic DNA of 21 varieties of *C. tetragonoloba*; (**b**) genetic relationship among the 21 clusterbean accessions as revealed by the UPGMA method in the Numerical Taxonomy System (NTSYS-pc) ver. 2.1

A total of 40 SSR markers were chosen randomly for validation in 21 clusterbean genotypes. The details of the SSR containing sequences and primers synthesized are provided in Supplementary Table S6. Out of 40 SSR primers, 28 SSR primers resulted in amplification in the target clusterbean varieties and were thus validated (Figure 5a). Two of the markers amplified multiple fragments (PCR products) while 26 showed single amplicons. Of the 28 SSRs, 13 were polymorphic with a significant polymorphism rate of 46.43%. The polymorphic information content (PIC) of the polymorphic markers ranged from 0.091 to 0.363 with an average of 0.21. The 21 genotypes were clustered into two distinct groups based on these markers (Figure 5b). To test the cross transferability of the markers, PCR was done with three genotypes each of pigeonpea and chickpea. However, no amplification could be found in any of the 28 primers in these two legume species. The FASTA sequences of predicted SSRs in HQ unigenes were BLAST searched against the EST sequences of *Cajanus cajan* (25,576 ESTs) and *Cicer arietinum* (52,788 ESTs), but no single hit was found with any EST sequence of these two legumes indicating the presence of uncharacterized SSRs unique to the clusterbean genome.

### 3.8. Database for Clusterbean Unigenes and Microsatellite Markers

The clusterbean unigenes database has all the 48,007 unigenes identified in the present study along with their details on transcript length, protein description, Blast2Go annotation, *E*-value, expression status in leaf, stem and flower tissues and the SSR motifs present in them. Searching unigenes by GO IDs, GO names, enzyme IDs, enzyme names, key words such as MYB, WRKY, Zinc ion binding, chloroplast (targeted) and the IDs assigned by us has been enabled in the database (Supplementary Figure S7). Separate tabs for retrieving information on the expression status of selected genes in individual tissues, in batch of 10 genes is given. Since SSR markers remain breeders’ choice, owing to the easy to genotype and locus specific nature of the markers, another tab for searching SSRs in unigenes has also been enabled. This information is also provided in the Supplementary Table S7.

## 4. Discussion

*C. tetragonoloba* is an annual legume crop and is a source of gum, food, fodder, and medicines [5,6,42]. It is a recently evolved and the only cultivated crop species among the 3 species of fabaceous genus *Cyamopsis* [43]. To augment the genomic resources and to facilitate whole genome assembly and marker development in this industrially and nutritionally important but orphan crop, we carried out transcriptome sequencing from leaf, shoot and flower tissues of clusterbean variety RG 936 in the present study.

From the ~150 million paired-end raw reads generated, we could retain ~145 million high-quality trimmed reads (22.16 Gb) despite stringent standards (quality score of 33). With 127,706 (179.50 Mb) transcripts and 48,007 (76.01 Mb) high-quality unigenes and 22.16 Gb raw reads, the depth of sequencing of the present study was in the range of 150–300x. Average length of contigs and N50 are the two important aspects to judge the quality of an assembly and on these both accounts, our assembly was better than the recently reported de novo transcriptomes across plant species such as *Onobrychis viciifolia, Nicotiana benthamiana* and leaf transcriptome of clusterbean where the N50 values were in the range of 1000–1400 bp and average lengths were around 700–780 bp [16,44,45]. In our study, N50 value was >2 kb with the average length of assembled transcript reaching 1376 bp which further increased to 1583 bp for unigenes. The average length and N50 value of our assembly was also found better than other closely related legumes with published transcriptomes, including *Cicer arietinum* [46,47], *Arachis hypogaea* [48], *Vigna radiata* [49,50] and *Trifolium pratense* [51]. Among the 48,007 high-quality unigenes, 63% (30,276) were more than 1 kb long and only 17.63% (8466) were less than 500 bp, which is again very high compared to the legume transcriptomes available in the public domain. The genome completeness of this transcriptome assembly was checked using CEGMA (Core Eukaryotic Genes Mapping Approach) pipeline [52] and the results showed that the transcriptome was complete to the tune of 98.79% with core eukaryotic genes (CEGs).

Of the 48,007 HQ unigenes of *C. tetragonoloba* transcriptome, 37,382 unigenes (77.87%) could be annotated even with a higher and more significant *E*-value cut-off of ≤ 1 × 10^−10^. Only 22.13% unigenes had no significant matches, which could be either because of the high cut-off or shorter transcript length. Moreover, these genes may represent the novel or clusterbean lineage specific genes or non-coding RNAs. From the cDNA libraries of clusterbean, 27% of the genes could not be annotated and could be non-coding RNAs [15]. BLAST results showed that clusterbean unigenes had the highest similarity with *Glycine max* (31.70%) which is consistent with the study by Tanwar et al. [16]. However, in the latter study the other species that showed higher similarity (6-15%) were *Phaseolus vulgaris*, *Cicer arietinum*, *Sphingomonas melonis* and *Medicago truncatula*. Though *Phaseolus vulgaris* (3.27%), *Cicer arietinum* (2.52%) showed similarity with clusterbean HQ genes in our study, the degree of similarity was much lower, while *Sphingomonas melonis* and *Medicago truncatula* did not ever feature in the top similarity species list. Rather, all the three species of Phaseoleae tribe of legumes, namely *Glycine max* followed by *Vigna angularis* (26.63%) *Cajanus cajan* (13.92%) showed the highest matches in our study. Moreover, the presence of only legumes in the high level of similarity list with clusterbean indicated good coverage of the homologous legume sequences in the assembly [51].

Coding capacity of the genomes is well captured when multiple tissues and multiple growth conditions are used for transcriptome sequencing. Since ESTs/transcripts from seed and leaf tissues are already reported, our transcriptome study with leaves, shoots and flowers is an improvement over the available resources [15,16]. After meta-analysis, it would be possible to identify tissue specific genes for leaves, flowers, seeds and shoots. 

Molecular markers identified from transcriptome-based studies are genic in nature and hence are expected to be more useful in molecular breeding applications. Transcriptome-based markers are helpful in contrast to the markers in non-transcribed regions owing to their high amplification rates and cross-species transferability [53]. Though SNPs are the markers of choice for understanding the trait architecture [54], for breeding application, SSRs and other gene/length polymorphism based markers are preferred. In the present work, one SSR per 8.75 kb in the HQ unigenes was identified which is in line with the earlier reports of one SSR per 7.31 kb [16] and one SSR per 7.9 kb [23] in clusterbean. Compared to other legumes, chickpea with one SSR per every 5.80 kb and common bean with one SSR per 4.70 kb, the frequency of SSRs in clusterbean seems to be lower [46,55].

Of the 40 randomly chosen SSRs, 28 could be validated by amplification. Such rates of successful amplification (70%) show that the majority of unigenes were precisely assembled. The failure of the remaining primer pairs to generate amplicons might be attributed to either long intervening introns or the location of primers across splice sites. This has also provided a novel set of genic SSRs to clusterbean research community. The cross-transferability studies with *Cajanus cajan* and *Cicer arietinum* failed to amplify SSRs even for a single primer pair. Following this, we subjected the complete set of FASTA sequences containing microsatellite markers and their flanking sequences to BLAST analysis with the two species and found no hits. This suggested that the clusterbean genome is unique and is quite diverse from the rest of the legumes including those belonging to the tribe Phaseoleae. Thus, further efforts would be required to generate multi-tissue transcriptomes, whole genome sequencing and useful molecular markers in this legume for breeding applications, evolutionary studies and understanding its genetic architecture.

## 5. Conclusions

The current study is the first report on multi-tissue developmental transcriptome from clusterbean and is third in a row after seed specific ESTs and leaf specific transcriptome. We have identified 48,007 high quality unigenes of which more than 98% have complete ORFs and 10,565 are clusterbean specific. Further, unigene sequence information and SSR markers have been provided in a database for easy access to researchers. Since clusterbean is an important crop in terms of gum and cosmetic industry, genomic and genetic information generated in the present study will serve as a platform for precise breeding applications.

## Figures and Tables

**Table 1:** Summary of the trimming results with Trimmomatic for each cDNA library sequenced.

Library/Sample	Number of Raw Reads (Paired)	Number of HQ Reads (Paired)	Number of HQ Reads (Un-Paired)	HQ Reads (Bases)	Average Length (HQ Paired Reads)
**Flower**	16,325,263	11,896,062	3,967,411	2,408,942,984	88.54
**Leaf**	41,575,280	30,676,700	9,701,552	6,192,249,152	88.91
**Shoot**	92,030,452	66,439,261	22,849,685	13,560,175,894	88.90
**Total**	149,930,995	109,012,023	36,518,648	22,161,368,030	

HQ: high quality.

**Table 2:** Transcriptome assembly and functional annotation of *Cyamopsis tetragonoloba.*

Assembly Statistics	Data
**Transcripts**	
Total Assembled	127,706 (179.50 Mb)
Average Length	1405.63 bp
GC%	39.22
≥1000 bp	64,606 (150.06 Mb)
≥5000 bp	2218 (138.75 Mb)
≥10,000 bp	53 (628.55 kb)
Largest Transcripts	16,940 bp
N50 Length	2263 bp
N75 Length	2931 bp
L50	26,460
L75	14,819
**Unigenes**	
Total Number	110,485 (152.13 Mb)
Average Length	1376.95 bp
Number of HQ Unigenes	48,007 (76.01 Mb)
Average Length (HQ Unigenes)	1583.43 bp
N50 Length (HQ Unigenes)	2179 bp
GC% (HQ Unigenes)	39.87
**Annotation of HQ Unigenes**	
Database Searched	Unigenes with significant hits
Against NCBI-Plant-Genes	37,382
Against SwissProt DB	28,905
Against Pfam	34,752
With Gene Ontology (GO) terms	30,020
With Enzyme Commission (EC) numbers	9984
All annotated transcript	37,442
With No Significant hit	10,565

**Table 3:** Statistics of simple sequence sepeats (SSRs) identified by MISA.

Features	Transcripts	HQ Unigenes
Total number of sequences examined	127,706	48,007
Total size of examined sequences (bp)	179,507,503	76,015,970
Total number of identified SSRs	17,593	8687
Number of SSR containing sequences	14,566	7047
Number of sequences containing more than 1 SSR	2430	1297
Number of SSRs present in compound formation	1137	590
Frequency of SSRs	1 SSR/10.20 kb	1 SSR/8.75 kb

## Supplementary Materials

The following are available online at www.mdpi.com/2073-4425/8/11/313/s1. Figure S1: Length distribution of HQ unigenes; Figure S2: GC content distribution of HQ unigenes; Figure S3: EC numbers, to categorize unigenes into 6 EC Classes; Figure S4. Validation of expression patterns of 7 randomly selected differentially expressed unigenes (based on FPKM values) using qRT-PCR to the show the similar patterns in both FPKM and qRT-PCR expression values, in blue and red color bar, respectively. The FPKM expression value of leaf tissue has been normalized with qRT-PCR. The respective unigene IDs are shown at the top and Y-axis represents relative expression values; Figure S5: Volcano and MA plots of DEGs for all 3 possible pair of tissue samples: Flower vs. Leaf, Flower vs. Shoot and Leaf vs. Shoot, respectively, with red dots as significant expression and black ones representing ‘no significant expression’ [FDR = False Discovery Rate; FC = Fold Change]; Figure S6: A clustered heatmap showing the Pearson correlation matrix for pairwise comparison between three tissue samples by comparing the complete transcriptome; Figure S7: Screenshot of ClustergeneDB, a database for retrieving information on the unigenes of clusterbean; Table S1: Assembly with different assembler; Table S2: Number of expressed genes for expression level of genes at different fold FPKM value; Table S3: Categorization of expressed genes; Table S4: Distribution of SSRs in different repeat classes in Transcripts; Table S5: Distribution of SSRs in different repeat classes in HQ unigenes; Table S6: The details of the SSR containing sequences which were used for validation; Table S7: Primer sequences of SSR markers identified from HQ unigenes.
