# Supplementary material for: High Quality Unigenes and Microsatellite Markers from Tissue Specific Transcriptome and Development of a Database in Clusterbean (Cyamopsis tetragonoloba, L. Taub)
Source: Genes (Basel). 2017 Nov 9;8(11):313. doi: 10.3390/genes8110313 (PMC5704226; doi:10.3390/genes8110313)
Supplement: Supplementary file 1 [file genes-08-00313-s001.zip › Supplementary/Supplementary figure legends.docx]

**Supplementary figure legends**

Supplementary Figure S1. Length distribution of HQ unigenes

Supplementary Figure S2. GC content distribution of HQ unigenes

Supplementary Figure S3. EC numbers, to categorize unigenes into 6 EC Classes

Supplementary Figure S4. Validation of expression patterns of 7 randomly selected differentially expressed unigenes (based on FPKM values) using qRT-PCR to the show the similar patterns in both FPKM and qRT-PCR expression values, in blue and red color bar, respectively. The FPKM expression value of leaf tissue has been normalized with qRT-PCR. The respective unigenes id are shown at the top and Y-axis represents relative expression values

Supplementary Figure S5. Volcano and MA plots of DEGs for all 3 possible pair of tissue samples: Flower *vs* Leaf, Flower *vs* Shoot and Leaf *vs* Shoot, respectively, with red dots as significant expression and black ones representing ‘no significant expression’ [FDR=False Discovery Rate; FC=Fold Change]

Supplementary Figure S6. A clustered heatmap showing the Pearson correlation matrix for pairwise comparison between three tissue samples by comparing the complete transcriptome

Supplementary Figure S7. Screenshot of ClustergeneDB, a database for retrieving information on the unigenes of cluster bean
