# Supplementary material for: High Quality Unigenes and Microsatellite Markers from Tissue Specific Transcriptome and Development of a Database in Clusterbean (Cyamopsis tetragonoloba, L. Taub)
Source: Genes (Basel). 2017 Nov 9;8(11):313. doi: 10.3390/genes8110313 (PMC5704226; doi:10.3390/genes8110313)
Supplement: Supplementary file 1 [file genes-08-00313-s001.zip › Supplementary/Supplementary tables.docx]

Supplementary Table S1. Assembly with different assembler.

|  | Contigs (>200) | Assembled Size (Mb) | Largest Contig Size(bp) | Average Contig size (bp) | N50 Size |
| --- | --- | --- | --- | --- | --- |
| CLC-30 | 111,011 | 77.55 | 16,673 | 698 | 1,098 |
| Galaxy-25 | 166,469 | 216.13 | 16,950 | 1298 | 2,315 |
| Trinity-25-norm30-cov2 | 127,706 | 179.50 | 16,940 | 1405 | 2,263 |
| Trinity-25-norm50-cov2 | 127,920 | 179.21 | 16,940 | 1401 | 2,250 |
| Spades-21 | 113,417 | 75.14 | 13,352 | 662 | 943 |
| Spades-33 | 105,784 | 80.52 | 16,011 | 761 | 1,219 |
| Spades-55 | 98,760 | 77.70 | 16,948 | 786 | 1,354 |

Supplementary Table S2: Number of expressed genes for expression level of genes at different fold FPKM value

| FPKM fold value | Shoot | Leaf | Flower | Total Expressed genes |
| --- | --- | --- | --- | --- |
| < 5-fold | 3,903 | 918 | 1,145 | 5,966 |
| 5-fold | 3,879 | 2,151 | 4,654 | 10,684 |
| 8-fold | 1,995 | 1,175 | 2,522 | 5,692 |
| 10-fold | 1,396 | 919 | 1,996 | 4,311 |
| 50-fold | 175 | 153 | 462 | 790 |

Supplementary Table S3: Categorization of expressed genes

| S. No. | Genes with FPKM ≥1 | Tissue-Type* | Expression Level (at 5 fold FPKM)# | | % of Total Expressed | Expression type |
| --- | --- | --- | --- | --- | --- | --- |
|  |  |  | Low | High |  |  |
| 1. | 28,089 | SLF | 38,252 | 11,732 | 64.65% | All |
| 2. | 39,228 | SF | 5,700 | 101 | 13.81% | Mixed |
| 3. | 30,851 | SL | 4,074 | 54 |  |  |
| 4. | 30,765 | FL | 739 | 10 |  |  |
| 5. | 56,367 | S | 3,903 | 3,879 | 21.54% | Tissue Specific |
| 6. | 55,351 | F | 1,145 | 4,654 |  |  |
| 7. | 38,349 | L | 918 | 2,151 |  |  |

*S=Shoot, F=Flower, L=Leaf; #Low: Genes with <5 FPKM or <5-fold FPKM value as compared with other. High: Genes with >5 FPKM or >5-fold FPKM value as compared with other tissues

Supplementary Table S4: Distribution of SSRs in different repeat classes in Transcripts

| Repeat Type\Number | 5 | 6 | 7 | 8 | 9 | 10 | >10 | Total | % of Repeat Types |
| --- | --- | --- | --- | --- | --- | --- | --- | --- | --- |
| Di | 0 | 3267 | 1848 | 1128 | 663 | 427 | 574 | 7907 | 44.94 |
| Tri | 5014 | 2144 | 869 | 354 | 56 | 51 | 136 | 8624 | 49.02 |
| Tetra | 587 | 156 | 32 | 28 | 5 | 0 | 3 | 811 | 4.61 |
| Penta | 96 | 31 | 1 | 3 | 1 | 0 | 1 | 133 | 0.76 |
| Hexa | 86 | 15 | 14 | 1 | 2 | 0 | 0 | 118 | 0.67 |
| Total | 5783 | 5613 | 2764 | 1514 | 727 | 478 | 714 | 17593 |  |
| % of Repeat Number | 32.87 | 31.90 | 15.71 | 8.61 | 4.13 | 2.72 | 4.06 |  |  |

Supplementary Table S5: Distribution of SSRs in different repeat classes in HQ unigenes

| Repeat Type\Number | 5 | 6 | 7 | 8 | 9 | 10 | >10 | Total | % of Repeat Types |
| --- | --- | --- | --- | --- | --- | --- | --- | --- | --- |
| Di | 0 | 1555 | 836 | 544 | 314 | 195 | 300 | 3744 | 43.10 |
| Tri | 2555 | 1080 | 453 | 204 | 30 | 32 | 86 | 4440 | 51.11 |
| Tetra | 269 | 79 | 15 | 16 | 3 | 0 | 3 | 385 | 4.43 |
| Penta | 47 | 12 | 1 | 2 | 0 | 0 | 0 | 62 | 0.71 |
| Hexa | 38 | 10 | 5 | 1 | 2 | 0 | 0 | 56 | 0.64 |
| Total | 2909 | 2736 | 1310 | 767 | 349 | 227 | 389 | 8687 |  |
| % of Repeat Number | 33.49 | 31.50 | 15.08 | 8.83 | 4.02 | 2.61 | 4.48 |  |  |
