# Supplementary figures and images for: High Quality Unigenes and Microsatellite Markers from Tissue Specific Transcriptome and Development of a Database in Clusterbean (Cyamopsis tetragonoloba, L. Taub)
Source: Genes (Basel). 2017 Nov 9;8(11):313. doi: 10.3390/genes8110313 (PMC5704226; doi:10.3390/genes8110313)

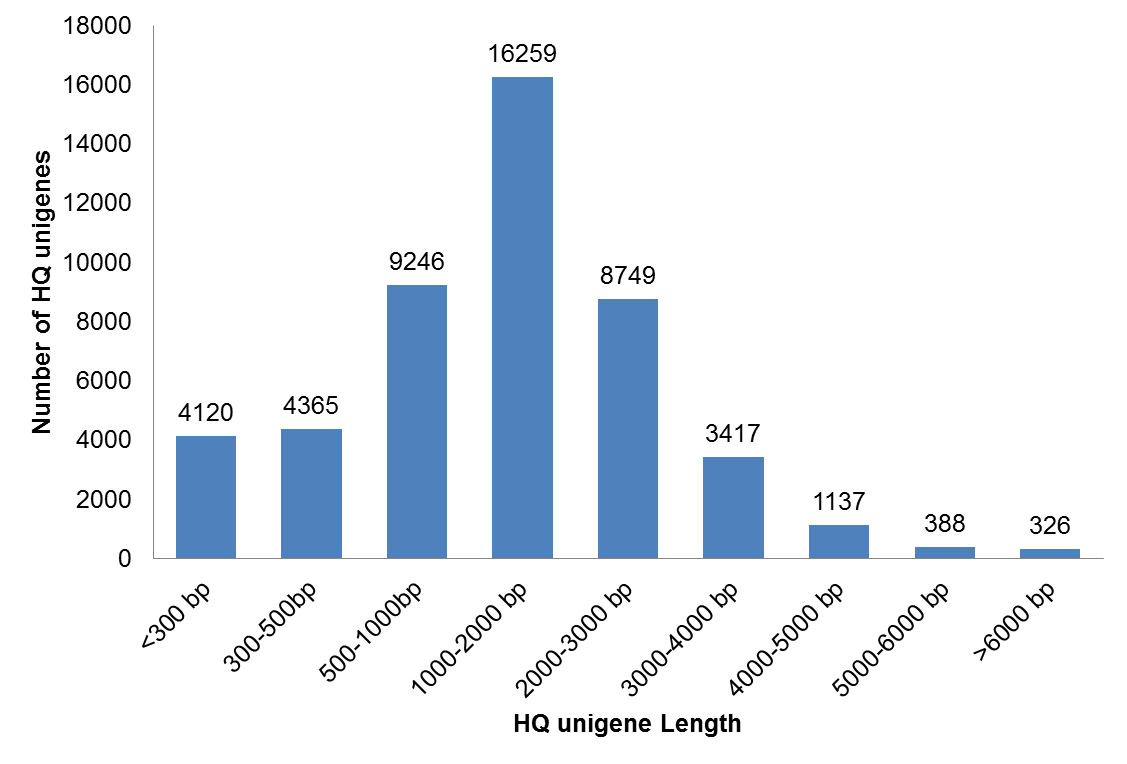

Supplement: Supplementary file 1 [file genes-08-00313-s001.zip › Supplementary/Fig S1.tif]

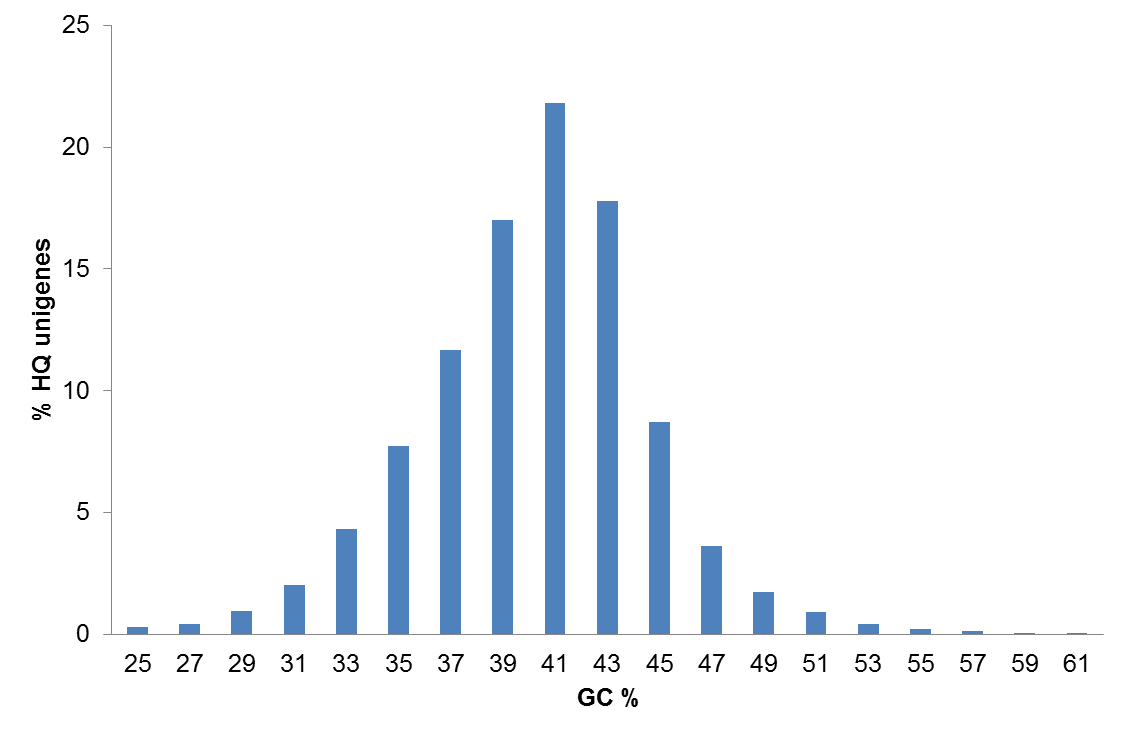

Supplement: Supplementary file 1 [file genes-08-00313-s001.zip › Supplementary/Fig S2.tif]

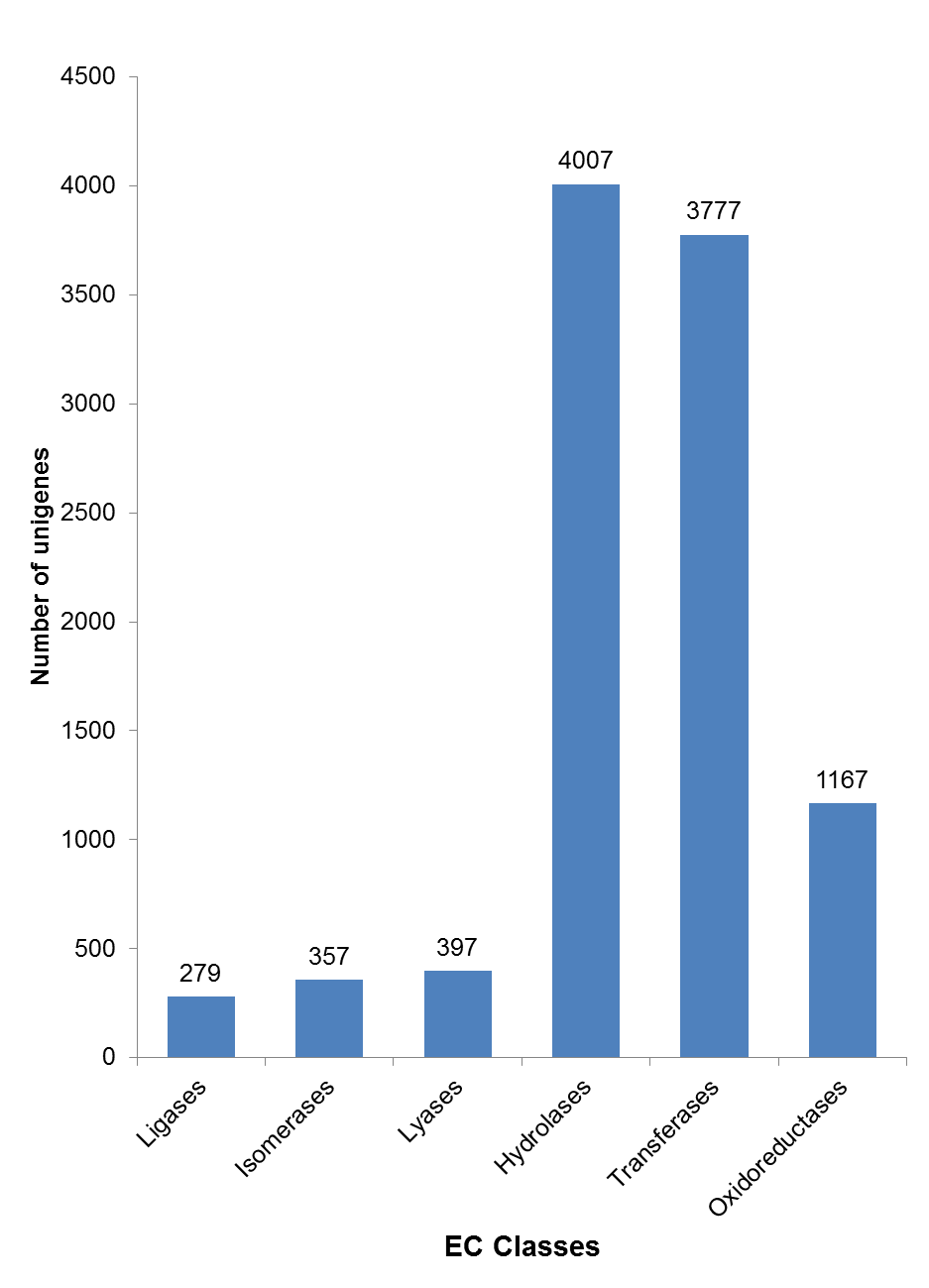

Supplement: Supplementary file 1 [file genes-08-00313-s001.zip › Supplementary/Fig S3.tif]

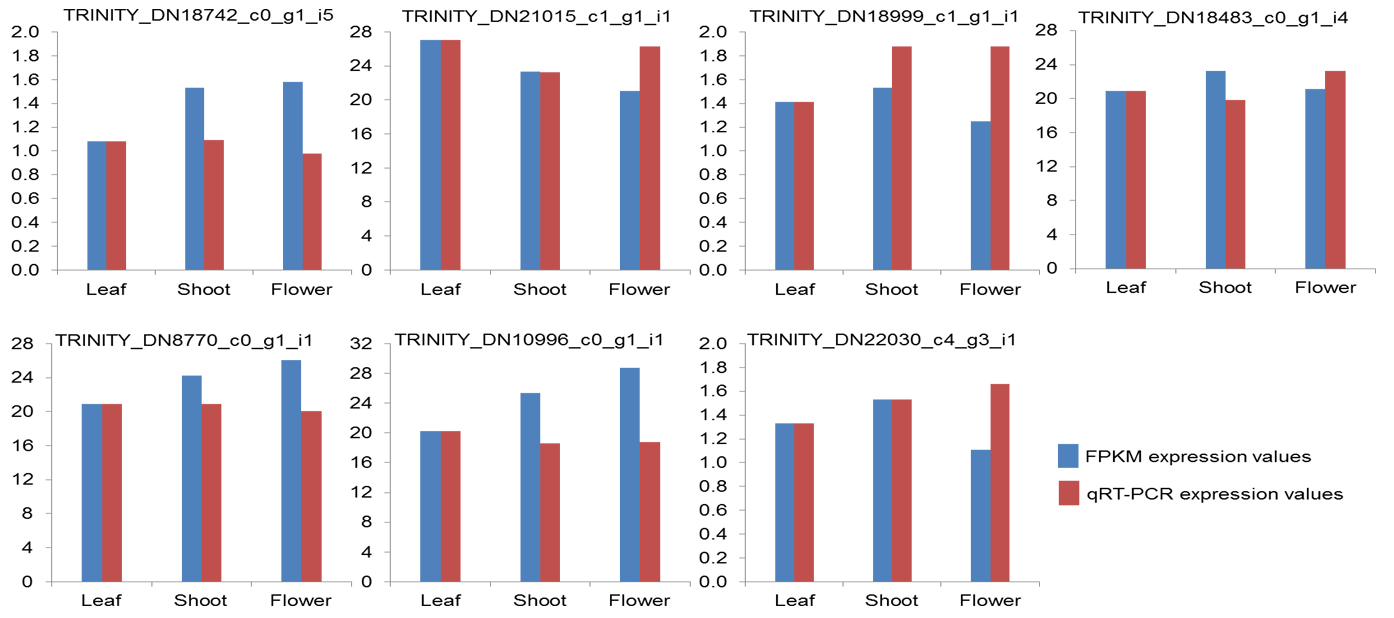

Supplement: Supplementary file 1 [file genes-08-00313-s001.zip › Supplementary/Fig S4.tif]

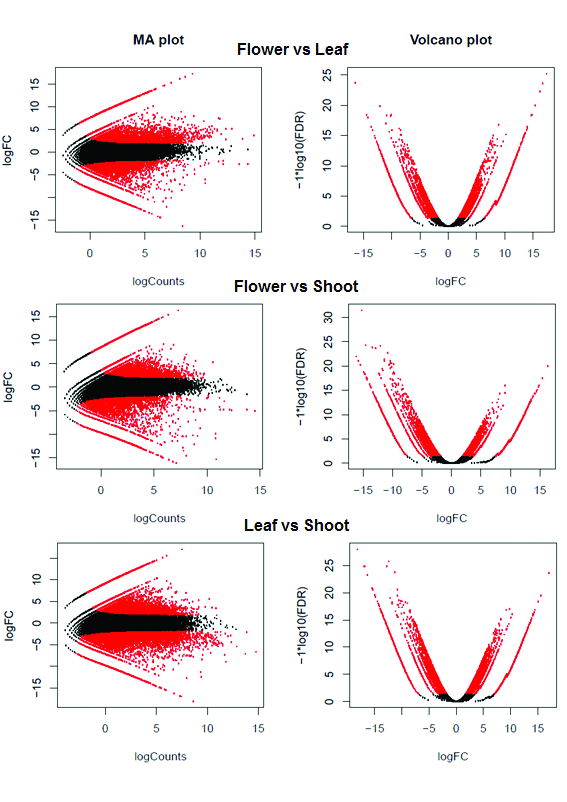

Supplement: Supplementary file 1 [file genes-08-00313-s001.zip › Supplementary/Fig S5.tif]

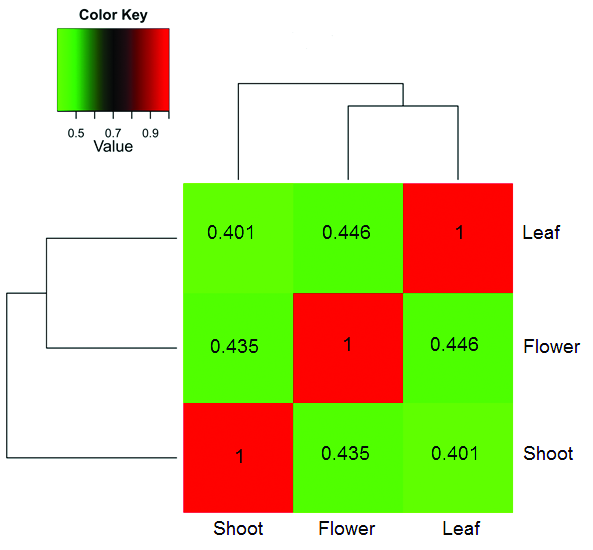

Supplement: Supplementary file 1 [file genes-08-00313-s001.zip › Supplementary/Fig S6.tif]

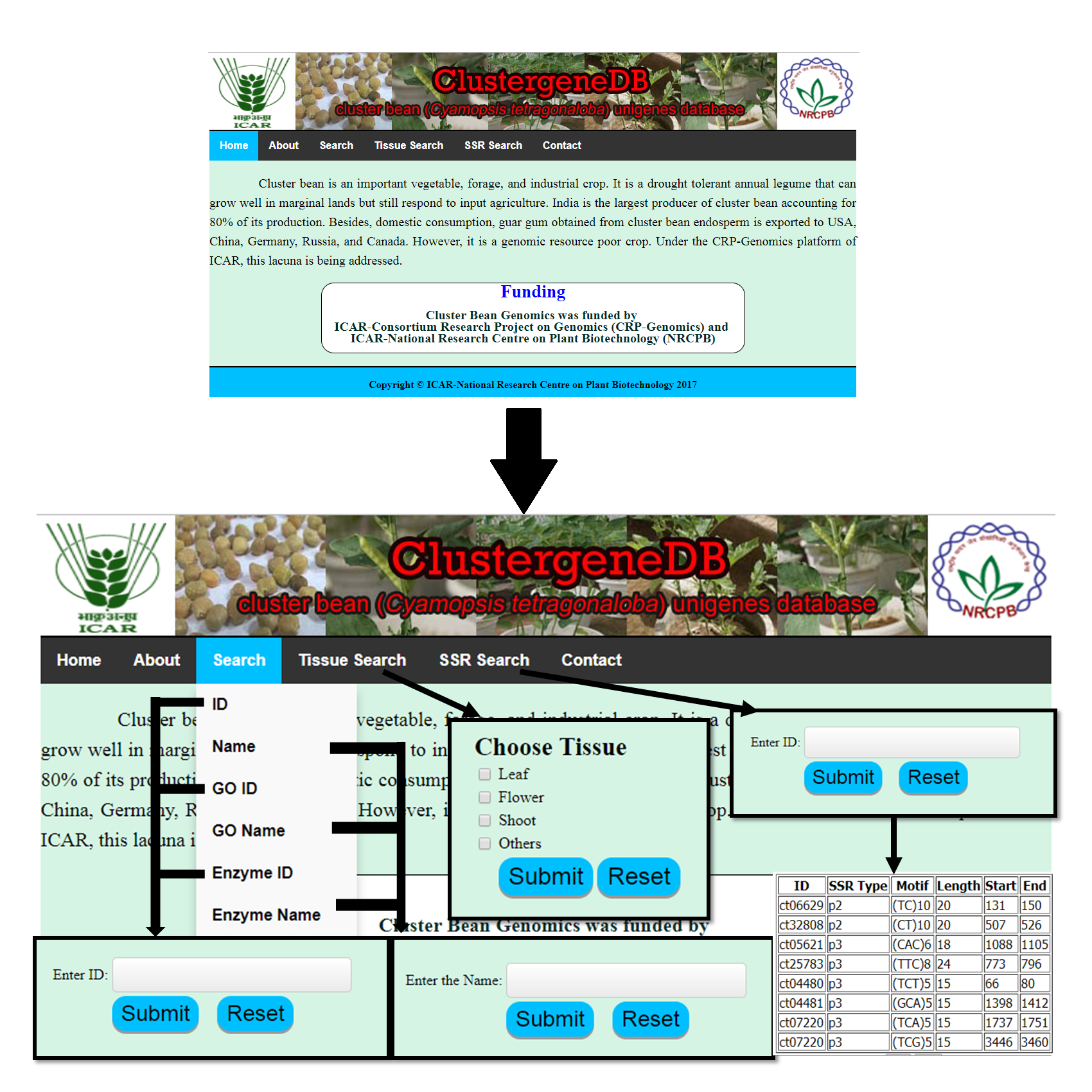

Supplement: Supplementary file 1 [file genes-08-00313-s001.zip › Supplementary/Fig S7.tif]
